# Post-induction residual time: a novel sensing phenomenon during induced ventricular fibrillation in subcutaneous ICD recipients

**DOI:** 10.3389/fcvm.2026.1949877

**Published:** 2026-09-24

**Authors:** Carlos Lopez, Ignacio Mondragon, Mónica Benjamin, Leandro Tomas, Eliana Aversa, Mauricio Abello, Juan Armentano, Javier Chaves

**Affiliations:** 1 Division of Cardiology, Hospital General de Agudos Dr. Cosme Argerich, Buenos Aires, Argentina; 2 Instituto Cardiovascular de Buenos Aires, Buenos Aires, Argentina; 3 Hospital de Pediatria Prof Dr. Juan P. Garrahan, Buenos Aires, Argentina; 4 Sanatorio San Lucas-San Isidro, Buenos Aires, Argentina; 5 Hospital General de Agudos Juan A. Fernandez, Buenos Aires, Argentina; 6 Sanatorio Finochietto, Buenos Aires, Argentina; 7 Sanatorio Guemes, Buenos Aires, Argentina; 8 Clinica y Maternidad Colon, Mar del Plata, Argentina

**Keywords:** ICD (implantable cardioverter-defibrillator), sensing, S-ICD, sudden cardiac death, ventricular fibrillation

## Abstract

**Background:**

Subcutaneous implantable cardioverter defibrillators (S-ICD) are extensively used in the primary and secondary prevention of sudden cardiac death. Ventricular fibrillation (VF) induction during implantation remains standard to confirm device functionality.

**Objective:**

To compare electrocardiographic (ECG) and technical variables between induced and spontaneous ventricular arrhythmias in S-ICD recipients.

**Methods:**

A retrospective observational study was performed on 10 consecutive patients with S-ICD. Variables included episode duration, sensing time, charge time, and shock impedance. For each patient, the implant induced VF episode was compared to at least one spontaneous event during follow-up.

**Results:**

The mean age was 36.4 years (range: 22–61); 2 patients were male. All patients received intermuscular S-ICD implants, with the lead placed in the left parasternal position in 7 patients and right parasternal in 3. Episode duration and sensing time were consistently shorter in spontaneous events, and shock impedance was lower. Charge time, however, was longer in spontaneous events (by 1.4 to 1.8 s).

**Conclusions:**

In this case series, spontaneous ventricular arrhythmic events in S-ICD patients demonstrated shorter sensing and therapy delivery times compared with induced ventricular fibrillation episodes. A measurable Post-Induction Residual Time (PRT) was identified in all induced episodes and was absent during spontaneous ventricular arrhythmias, suggesting that 50-Hz-induced VF may not fully reproduce spontaneous arrhythmic events from the perspective of device sensing performance. These findings support the hypothesis that part of the delay observed during implant testing may be related to the induction process itself rather than to the intrinsic sensing capabilities of the S-ICD. Further studies are warranted to confirm these observations and to evaluate alternative VF induction strategies in future device platforms. Future integration of S-ICD systems with leadless pacing technologies may provide alternative ventricular fibrillation induction strategies, including T-wave shock delivery during either sensed intrinsic rhythm or leadless-paced rhythm. Such approaches could potentially avoid the occurrence of Post-Induction Residual Time (PRT) and may provide a model that more closely reproduces spontaneous ventricular fibrillation than current 50-Hz induction techniques.

## Introduction

The subcutaneous implantable cardioverter-defibrillator (S-ICD) is an established alternative to transvenous systems for selected patients requiring defibrillation therapy. A key step in the implant procedure involves inducing ventricular fibrillation (VF) to validate the device's detection and therapeutic response. However, it remains unclear whether induced arrhythmias accurately reflect the system's performance during spontaneous episodes (1–5).

This study aimed to analyze the differences in detection times for induced arrhythmias compared with spontaneous episode detection times, as well as variables such as shock impedance and charging time, in a series of patients with stored subcutaneous ECG data.

## Methods

An observational case series was conducted in 10 consecutive patients who received a Boston Scientific EMBLEM MRI S-ICD for secondary prevention of sudden cardiac death. Implantation was intermuscular in all cases. Data were obtained from clinical records and stored device information. Stored electrocardiographic recordings from induced and spontaneous ventricular arrhythmias were reviewed in all patients.

The main variables analyzed included total episode duration, sensing time (from arrhythmia onset to device detection), charging time, and shock impedance. In addition, we evaluated a novel parameter termed Post-Induction Residual Time (PRT). Post-Induction Residual Time (PRT) was defined as the interval between termination of the 50-Hz ventricular fibrillation induction burst and the first valid ventricular sensing marker recognized by the S-ICD detection algorithm. PRT was measured in all induced episodes and was not observed in spontaneous ventricular arrhythmias.

## Statistical analysis

Descriptive statistics were performed using IBM SPSS Statistics v26 and GraphPad Prism v9. Continuous variables were expressed as mean ± standard deviation (SD), categorical data are expressed as numbers (percentage), and paired comparisons between induced and spontaneous events were analyzed using the Wilcoxon signed-rank test. A *p*-value <0.05 was considered statistically significant.

## Results

The analysis included patients with idiopathic VF (60%), idiopathic dilated cardiomyopathy (30%), and Brugada syndrome (10%), with one patient having a history of previous infection. (Table 1).

**Table 1:** Baseline demographic characteristics of included participants.

Baseline demographic characteristics of included participants					
Variable	n (or mean)	%	IC 95%^b^		mean std
Sex					
Male	2	20%	3%	56%	———
Female	8	80%	44%	97%	———
*age*	36,4	———	26,12	46,67	4,54
Diagnostic					
IVF	6	60%	26%	88%	———
IDC	3	30%	7%	65%	———
Brugada	1	10%	0%	45%	———
Medication					
amiodarone^a^	3	30%	7%	65%	———
no drugs	7	70%	35%	93%	———
Indication					
secondary	10	100%	69%	100%	———
Implant type					
intermuscular	10	100%	69%	100%	———

a Patients with IDC.

b Confidence intervals under Clopper Pearson for all variables, except age (t-interval for the mean).

The mean age was 36.4 years (range: 22–61), and 2 patients were male. The average implantation time was 59.3 min (range: 38–75 min).

Lead position was left parasternal in 7 cases and right parasternal in 3 cases. Vector configurations included 2/3 sensing vectors in 7 patients and 3/3 in 3 patients. Overall, a measurable post-induction interval was present in all induced episodes and absent during spontaneous ventricular arrhythmias.

The main variables analyzed included total episode duration, sensing time (from onset to detection), charging time, and shock impedance.

Total event duration: Decreased on spontaneous episodes by 2.94 s (95% CI [−4.967; −0.913]) **(**Figure 1**).**

**Figure 1:**
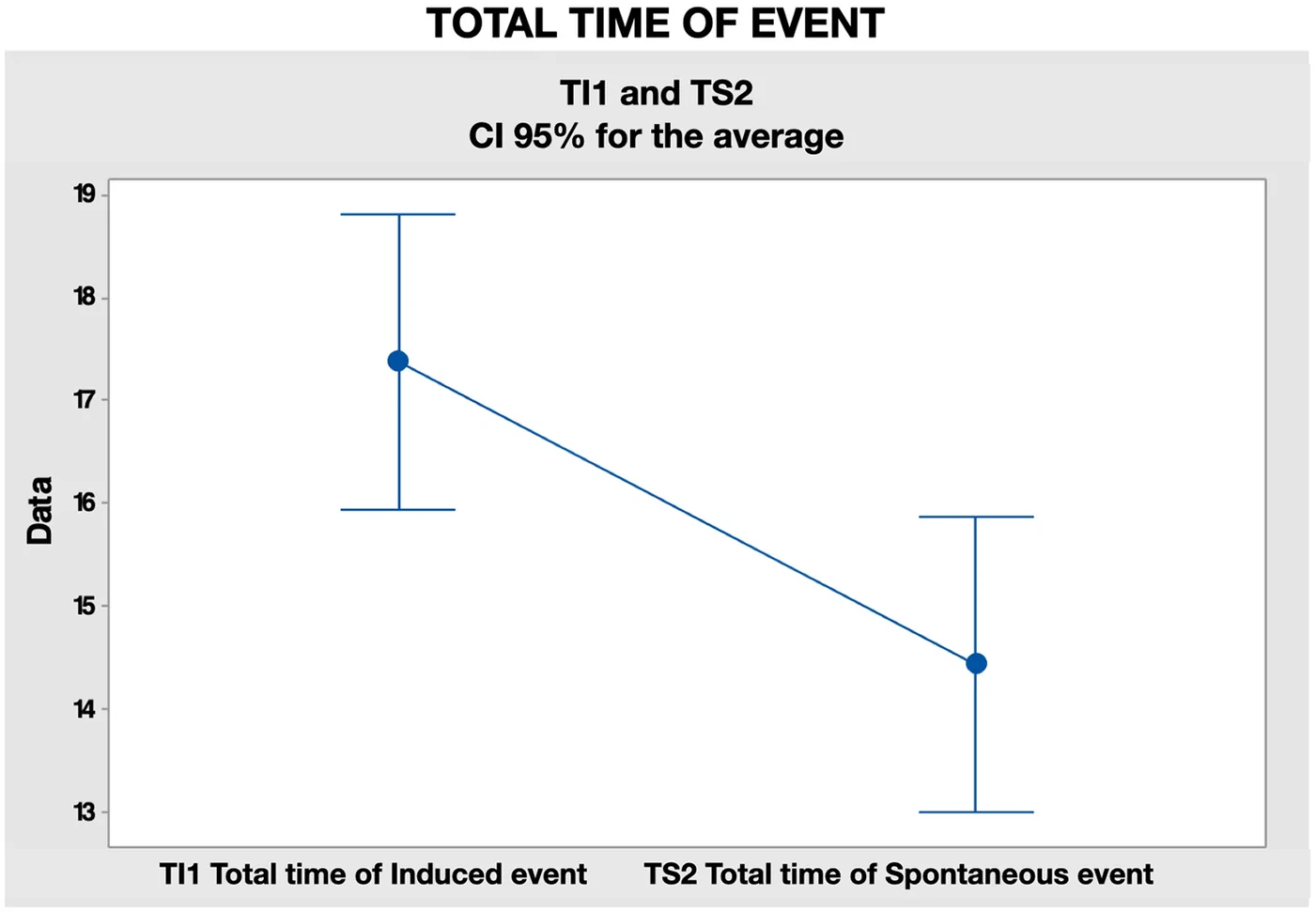
With a *p*-value of less than 1%, a significant difference is observed in the total mean times of induced and spontaneous events (spontaneous events are statistically smaller).

Sensing time: Decreased on spontaneous VF episodes by 3.1 s (95% CI [−4.824; −1.376]).

Charging time: Longer during spontaneous episodes by an average of 0.71 s, although the difference was not statistically significant.

Shock impedance: Lower during spontaneous events, suggesting improved current delivery dynamics; however, impedance did not show a significant difference (Figure 2).

**Figure 2:**
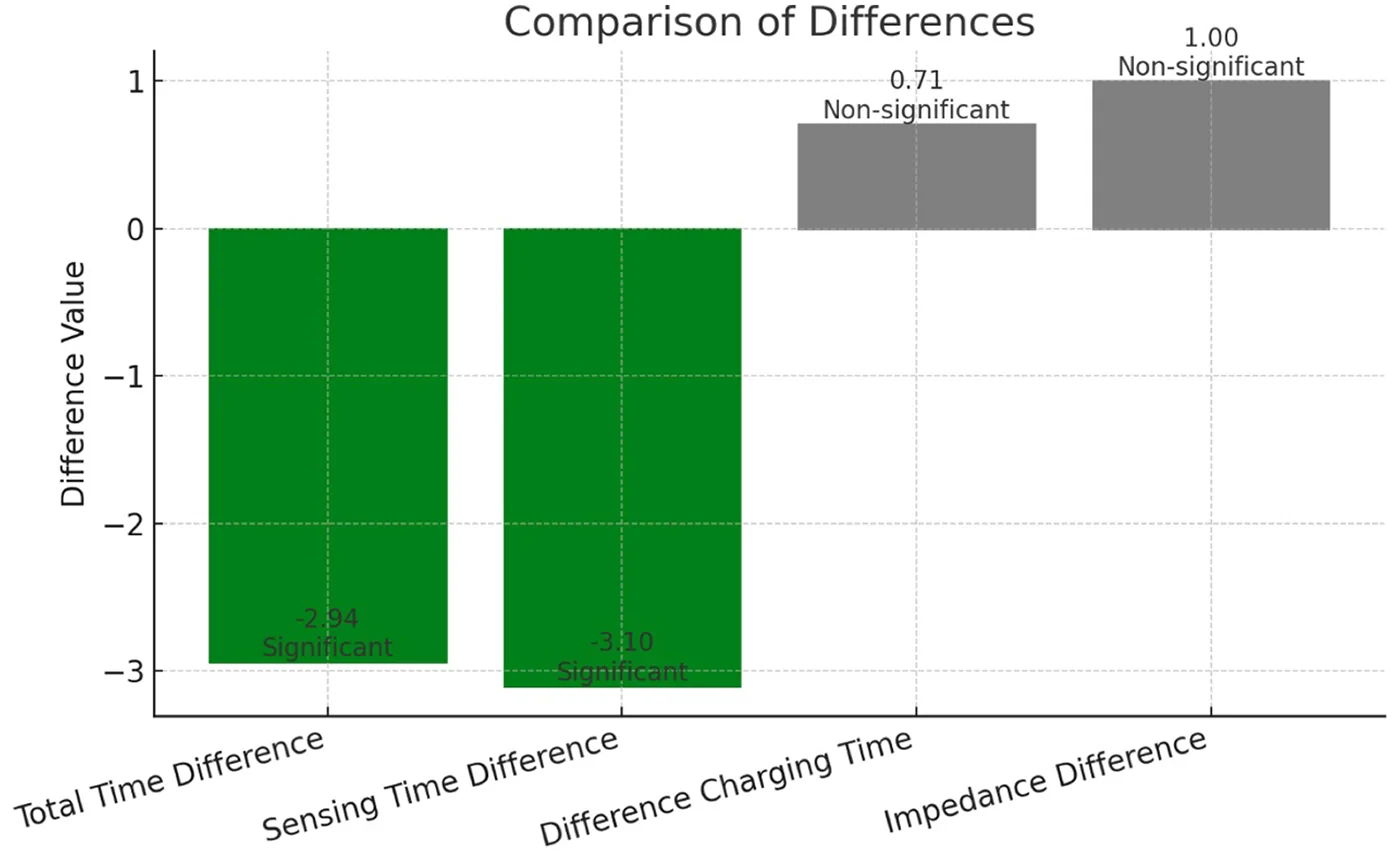
The figure graph presents a comparison of differences in four key variables.

These findings suggest improved device efficiency, with significant reductions in both total detection and sensing times during spontaneous episodes. However, differences in episode timing may also be influenced by factors beyond sensing/detection alone, including the context and therapy parameters applied during spontaneous events versus induction testing.

## Discussion

This preliminary analysis suggests that spontaneous ventricular arrhythmias may be detected and treated more efficiently by S-ICD than induced episodes. The observed post-induction interval after 50-Hz induction indicates that VF induced by an alternating-current protocol may not fully reproduce spontaneous VF from the perspective of device sensing dynamics. Importantly, this observation should not be interpreted as a device malfunction; rather, it reflects an outcome shaped by the induction procedure and the shared sensing/detection context across induced cases. (Figure 3).

**Figure 3:**
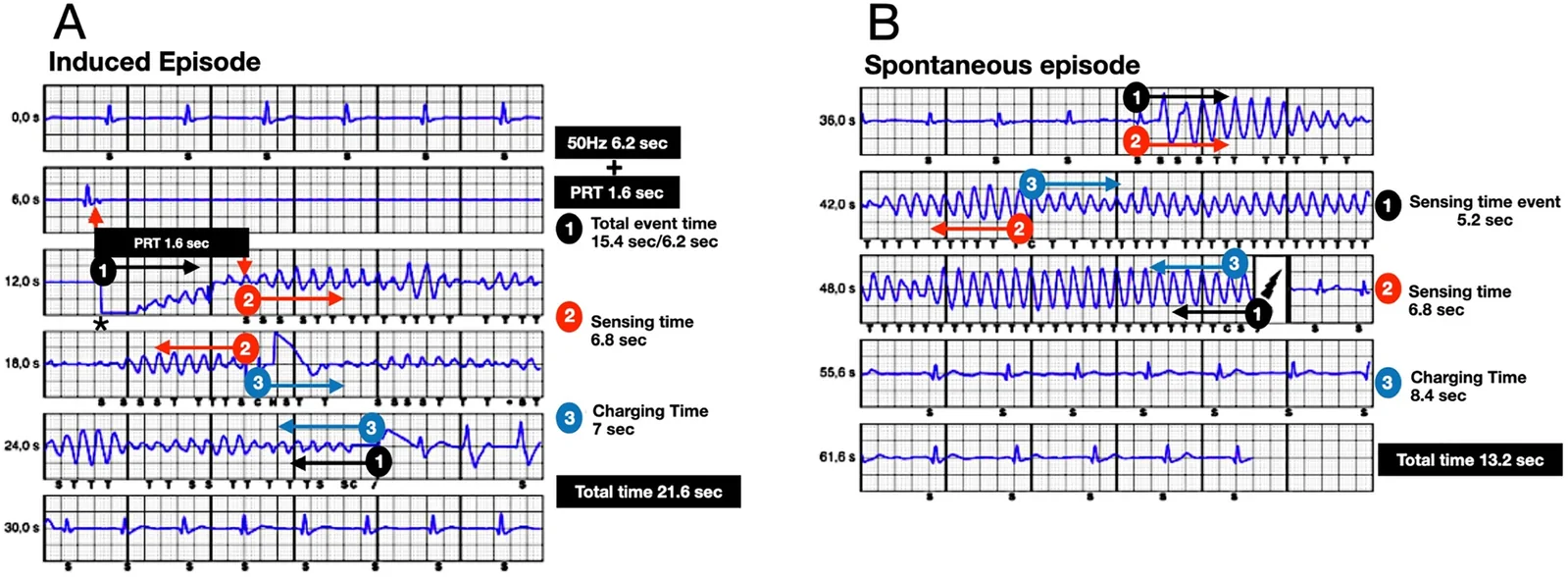
**(A)** VF event induced with 50 Hz with a post-induction time (PRT) of 1.6 s and a total episode time of 21.6 s **(B)** Spontaneous event in the same patient performing daily tasks, which, being an episode of spontaneous arrhythmia, does not have a PRT.

A key mechanistic interpretation is that the 50-Hz induction process may transiently affect sensing recovery, creating a brief interval before stable ventricular sensing is re-established. Consistent with this, the interval was not present during spontaneous ventricular arrhythmias, supporting the hypothesis that it is related to induction conditions rather than intrinsic differences in spontaneous VF physiology.

These results have methodological implications for how induction findings are extrapolated to real-world sensing/detection performance. This limitation should be considered alongside prior work describing the constraints of VF induction protocols and their relationship to device performance metrics (6, 7). In particular, evidence from the PRAETORIAN-DFT trial highlights that V*F* testing and induction strategies are tightly linked to how performance endpoints are operationalized and should be interpreted within the context of the testing paradigm (8). Previous S-ICD studies and registries have demonstrated the clinical performance of the system across different patient populations and clinical settings (9–12); however, these studies were not specifically designed to compare sensing dynamics between induced and spontaneous ventricular arrhythmias.

Despite the small sample size, these findings warrant further investigation in larger prospective studies. Future modular systems integrating leadless pacing technologies may enable alternative VF induction strategies, including T-wave shock induction, potentially reducing or avoiding the post-induction interval and providing a model that more closely resembles spontaneous ventricular fibrillation.

## Conclusions

In this case series, spontaneous ventricular arrhythmic events in S-ICD patients demonstrated shorter sensing and therapy delivery times compared with induced ventricular fibrillation episodes. A measurable post-induction interval (PRT) was identified in all induced episodes and was absent during spontaneous ventricular arrhythmias, suggesting that 50-Hz-induced VF may not fully reproduce spontaneous arrhythmic events from the perspective of device sensing dynamics.

These findings support the hypothesis that part of the delay observed during implant testing may be related to the induction process itself rather than intrinsic sensing capabilities of the S-ICD. Further studies are warranted to confirm these observations and to evaluate alternative VF induction strategies in future device platforms.

Future integration of S-ICD systems with leadless pacing technologies may provide alternative VF induction strategies, including T-wave shock delivery on either sensed intrinsic rhythm or leadless-paced rhythm. Such approaches could potentially avoid the post-induction interval and may provide a model that more closely resembles spontaneous ventricular fibrillation than current 50-Hz induction techniques.

## Data Availability

The original contributions presented in the study are included in the article/Supplementary Material, further inquiries can be directed to the corresponding author.
